# Performance of BinaxNOW G6PD Deficiency Point-of-Care Diagnostic in *P. vivax*-Infected Subjects

**DOI:** 10.4269/ajtmh.14-0298

**Published:** 2015-01-07

**Authors:** Lyda Osorio, Nick Carter, Preetam Arthur, Germana Bancone, Sowmya Gopalan, Sandeep K. Gupta, Harald Noedl, Sanjay K. Kochar, Dhanpat K. Kochar, Srivicha Krudsood, Marcus V. Lacerda, Alejandro Llanos-Cuentas, Ronnatrai Rueangweerayut, Ramadurai Srinivasan, Moritz Treiber, Jörg J. Möhrle, Justin Green

**Affiliations:** Epidemiology and Population Health Research Group (GESP), School of Public Health, Universidad del Valle, Cali, Colombia; Clinical Research and Development Fellowship, World Health Organization/Special Program for Research and Training in Tropical Diseases (WHO/TDR) at GlaxoSmithKline (GSK), Uxbridge, Middlesex, United Kingdom; GlaxoSmithKline Research and Development, Uxbridge, Middlesex, United Kingdom; Department of Medicine, Sri Ramchandra Medical College and Research Institute, Porur, Chennai, Tamil Nadu, India; Shoklo Malaria Research Unit, Mae Sot, Thailand; MV Hospital and Research Centre, Lucknow, Uttar Pradesh, India; Institute of Specific Prophylaxis and Tropical Medicine, Medical University of Vienna, Vienna, Austria; Sardar Patel Medical College, Bikaner, Rajasthan, India; Department of Medicine, Rajasthan University of Health Sciences Medical College, Jaipur, India; Faculty of Tropical Medicine, Mahidol University, Bangkok, Thailand; Fundação de Medicina Tropical Doutor Heitor Vieira Dourado, Manaus, Amazonas, Brazil; Instituto de Medicina Tropical, Alexander von Humboldt, Universidad Peruana Cayetano Heredia, Lima, Peru; Mae Sot Hospital, Mae Sot, Tak Province, Thailand; Medicines for Malaria Venture, Geneva, Switzerland

## Abstract

Accurate diagnosis of glucose-6-phosphate dehydrogenase (G6PD) deficiency is required to avoid the risk of acute hemolysis associated with 8-aminoquinoline treatment. The performance of the BinaxNOW G6PD test compared with the quantitative spectrophotometric analysis of G6PD activity was assessed in 356 *Plasmodium vivax-*infected subjects in Brazil, Peru, Thailand, and India. In the quantitative assay, the median G6PD activity was 8.81 U/g hemoglobin (range = 0.05–20.19), with 11 (3%) subjects identified as deficient. Sensitivity of the BinaxNOW G6PD to detect deficient subjects was 54.5% (6 of 11), and specificity was 100% (345 of 345). Room temperatures inadvertently falling outside the range required to perform the rapid test (18–25°C) together with subtlety of color change and insufficient training could partially explain the low sensitivity found. Ensuring safe use of 8-aminoquinolines depends on additional development of simple, highly sensitive G6PD deficiency diagnostic tests suitable for routine use in malaria-endemic areas.

## Background

The public health importance of *Plasmodium vivax* malaria is now more widely recognized as renewed targets for malaria elimination are defined.^1^ The capability of *P. vivax* to relapse from a liver stage known as the hypnozoite weeks or months after an initial infection poses a challenge for the estimation of its transmission burden and control efforts. At present, the 8-aminoquinoline primaquine is the only drug recommended by the World Health Organization (WHO) to treat *P. vivax* hypnozoites, but it is contraindicated in subjects with severe glucose-6-phosphate dehydrogenase (G6PD) deficiency. Mild G6PD-deficient subjects are recommended a dose of 0.75 mg base/kg one time per week for 8 weeks instead of the standard dose of 0.25–0.5 mg base/kg one time per day for 14 days.^2^ However, in a clinical setting, these differential dosages can only be implemented if G6PD deficiency status is known. Tafenoquine, a primaquine analog in late-state development, is likely to be contraindicated in G6PD-deficient subjects, with a cutoff as yet to be determined.^3^ The facts that the geographical distribution of malaria and G6PD deficiency is shown to overlap and that *Plasmodium*-infected subjects, therefore, might be exposed to high doses of 8-aminoquinolines highlight the need to assess the status of G6DP in the population to ensure the safe use of these drugs.^4^,^5^

G6PD catalyzes the oxidation of glucose-6-phosphate to 6-phosphogluconolactone with reduction of nicotinamide adenine dinucleotide phosphate (NADP) to reduced nicotinamide-adenine dinucleotide phosphate (NADPH), which is essential to protect cells from oxidative stress. Red blood cells (RBCs) are particularly dependent on G6PD, and thus, deficiency is commonly manifested clinically as hemolysis triggered by exogenous factors, such as infections, drugs (e.g., 8-aminoquinolines), and in some cases, fava beans and selected other foods.^6^ G6PD deficiency occurs as a result of gene mutations located in the X chromosome. Therefore, men are either normal or deficient, whereas women are normal if both X chromosomes are unaffected, deficient if both copies of the X chromosome are affected, or heterozygous if only one copy of the X chromosome is affected. Heterozygous women can show a wide spectrum of phenotypes, because they will have a variable mixture of normal and deficient cells because of lyonisation.^7^ This group is the most difficult to diagnose by phenotypic tests but remains at risk of hemolysis.

The quantitative spectrophotometric analysis of NADPH production is considered as the reference test to diagnose G6PD deficiency.^8^,^9^ It can be performed in hospital laboratories in endemic countries that have the required infrastructure. Nevertheless, this is not the case in most malaria-endemic regions, where infected patients are seen in primary care without the needed laboratory facilities, and therefore, a much simpler test is required. Classical methods, such as the methemoglobin reduction test, the fluorescent spot test, the methylene blue reduction test, and other dye discoloration tests, have been used in the field.^6^ More recently, extensions to these methods have been reported that are easier to use.^10^,^11^ However, they are not widely adopted for routine use, because they may take several hours to give results, require a cold chain, specialized reagents, or equipment, are susceptible to environmental conditions, or are difficult to interpret.^12^ Two chromatographic rapid diagnostic commercially available tests, BinaxNOW G6PD (Alere Inc., Waltham, MA) and CareStart G6PD (CareStar Inc., Cincinnati, OH), have reported sensitivities up to 98% (95% confidence interval [95% CI] not reported) and 68% (95% CI = 58–77) and specificities of 98% (95% CI not reported) and 100% (95% CI = 99–100), respectively.^13^,^14^ However, they have not yet been assessed in malaria-infected subjects whose G6PD status can be confounded by anemia and malaria-induced hemolysis.^15^ Therefore, we assessed the performance of the BinaxNOW G6PD as a potential screening test for G6PD deficiency in *P. vivax*-infected subjects before treatment with 8-aminoquinolines.

## Methods

### Study design and population.

This was a cross-sectional study conducted in subjects screened within a multicenter clinical trial testing the efficacy of 8-aminoquinolines primaquine and tafenoquine for the radical cure of *P. vivax* malaria in Brazil, Peru, Thailand, and India (Clinicaltrials.gov ID NCT01376167), and additional information is reported elsewhere.^3^ Consecutive subjects with a positive smear for *P. vivax* who were 16 years old or older attending the Tropical Medicine Foundation of Amazonas Dr. Heitor Viera Dourado in Manaus, Brazil; Clinica Selva Amazonica in Iquitos, Peru; Mae Sot General Hospital in Tak, Thailand; the Faculty of Tropical Medicine, Mahidol University in Bangkok, Thailand; Sri Ramachandra Medical College and Research Institute in Chennai, India; Sardar Patel Medical College and AG Hospitals in Bikaner, India; MV Hospital and Research Center in Lucknow, India; and Krishna Institute of Medical Sciences in Secunderabad, India between September 19, 2011 and October 1, 2012 who gave written informed consent to participate were screened for G6PD deficiency as part of the entry criteria for the main study. The study was approved by both the local and national ethical committees of the participating institutions and regulatory agencies of the participating countries.

### Procedures.

Venous blood samples (4 mL) were collected in tubes containing ethylenediaminetetraacetic acid (EDTA) and processed within 6 hours with the BinaxNOW G6PD test. The tests were performed according to the manufacturer's instructions. Briefly, 10 μL each blood sample was mixed in an individual vial containing 70 μL lysis reagent (Tris buffer with detergent and red dye) before 50 μL lysed blood was transferred to the test device. The results were read after 7 minutes by an experienced laboratory technician at each study site. The procedure was done in a controlled temperature environment and protected from direct light. A G6PD-normal (negative) result was interpreted when a distinct color change to black/brown was observed, and a G6PD-deficient (positive) result was interpreted if there was no color change. Training to perform the BinaxNOW G6PD was not provided, and sites were asked to use the test according to the package insert. The remaining blood samples were stored at 4°C until they were processed at the site within 24 hours with the quantitative spectrophotometric analysis of G6PD as per the Trinity Biotech assay (345) in Bangkok, Thailand and India, the Pointe Scientific Inc. assay (G7583) in Brazil and Peru, or the WHO method in Mae Sot, Thailand.^16^ The reference tests were done by the local laboratory technicians using semiautomatic spectrophotometers with a temperature-controlled cuvette compartment (Shimadzu 1800 UV-Vis). Briefly, a reaction mixture containing 1.5 mM NADP, 12 mM maleimide (an inhibitor of 6-phosphogluconate dehydrogenase), buffer, stabilizer, and 0.05% volume/volume Triton X-100 as the lysing agent was prepared locally and stored at 4°C; 10 μL each blood sample was gently mixed with 1 mL reaction mixture and left to stand for 10 minutes at room temperature (range = 15–30°C) before adding 2 mL enzyme substrate (1.05 mM glucose-6-phophate, buffer, magnesium salt, and sodium azide as a preservative) and aliquoting in three test cuvettes, which were placed in the cuvette compartment at a constant 37°C (30°C in Thailand) to reach thermal equilibrium. Absorbance of samples at 340 nm was read at 300 and 600 seconds using the software UV PROBE (Shimadzu Inc.). The differences in the 300- and 600-seconds absorbance measurements divided by five yielded the G6PD activity per minute. The final result was the mean G6PD activity of the triplicates (duplicates in Bangkok) and reported as units of enzyme per gram of hemoglobin (Hb) when adjusted by volume and Hb concentration of the sample. Samples were appropriately temperature-corrected to 37°C. The Hb concentration of each sample was measured before the reference test using locally available semiautomated machines. For quality control, G6PD-lyophilized normal and deficient controls were included each time that the reference tests were performed. The control samples were purchased from Sigma Chemicals US (G6888 [normal controls] and G5888 [deficient controls]).

### Statistical analyses.

Data were entered in the data manager (Timaeus; Cmed, Horsham, United Kingdom) at each study site and analyzed using R. Participants with interpretable results in both the reference and BinaxNOW G6PD tests were included in the analyses. The study population and the reference test results were described using relative frequencies, medians, or means with corresponding ranges or SDs as appropriate. First, the frequency distributions of the quantitative G6PD activity were drawn for men and women separately. Second, quantitative G6PD activity results for the reference test were categorized following WHO guidelines as normal or deficient using as cutoff point of 60% of the median activity observed in the corresponding population.^6^ The reference G6PD activity value for each study site was obtained previously in a sample of 36 normal adult male subjects with Hb concentration ≥ 12 g/dL and reticulocyte count ≤ 2.5% (Table 1). Sensitivity, specificity, predictive values, and likelihood ratios of the BinaxNOW G6PD against the reference test were obtained with their corresponding 95% CIs.

## Results

**Figure 1.**
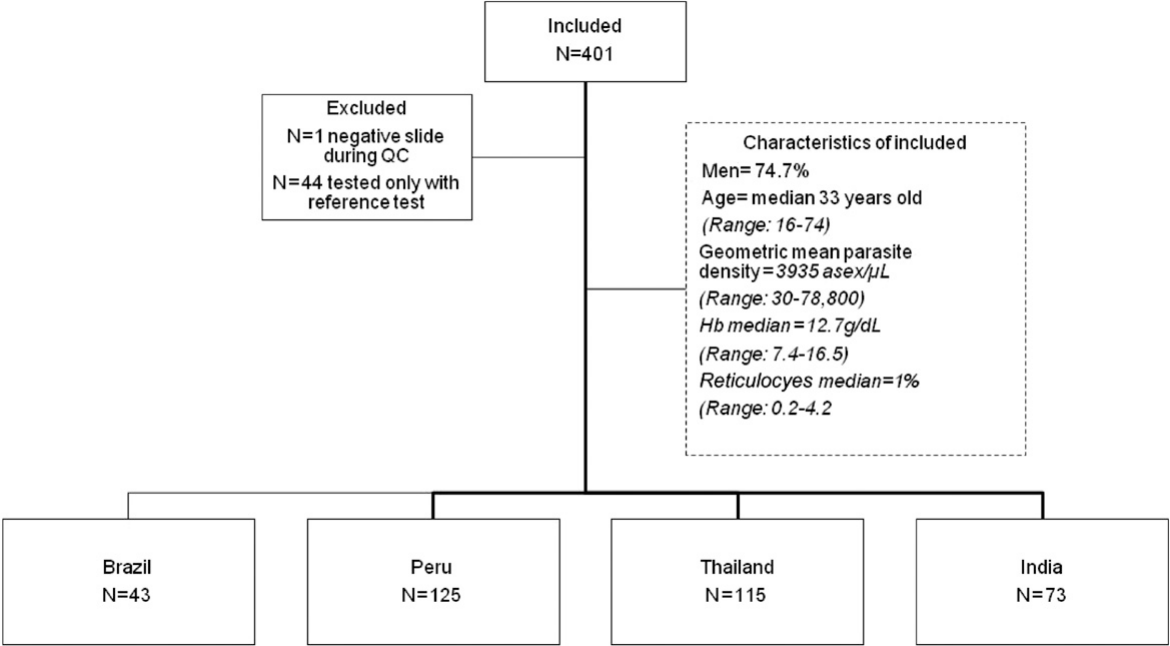
Diagram of study participants. QC = quality control.

**Figure 2.**
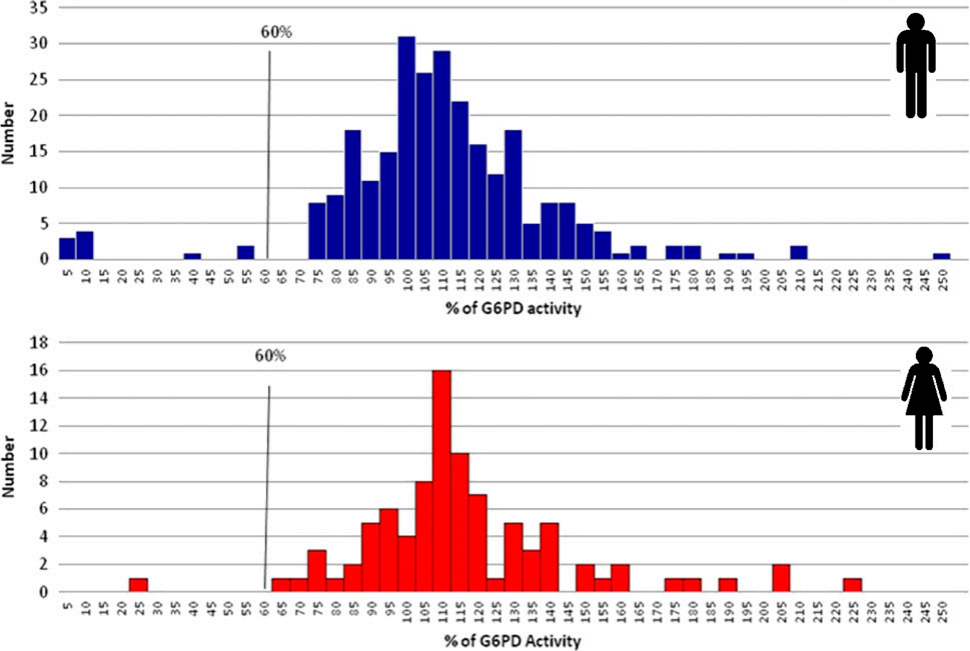
Distribution of G6PD activity (percentage of the normal value in the reference population) according to the quantitative assay by sex.

**Figure 3.**
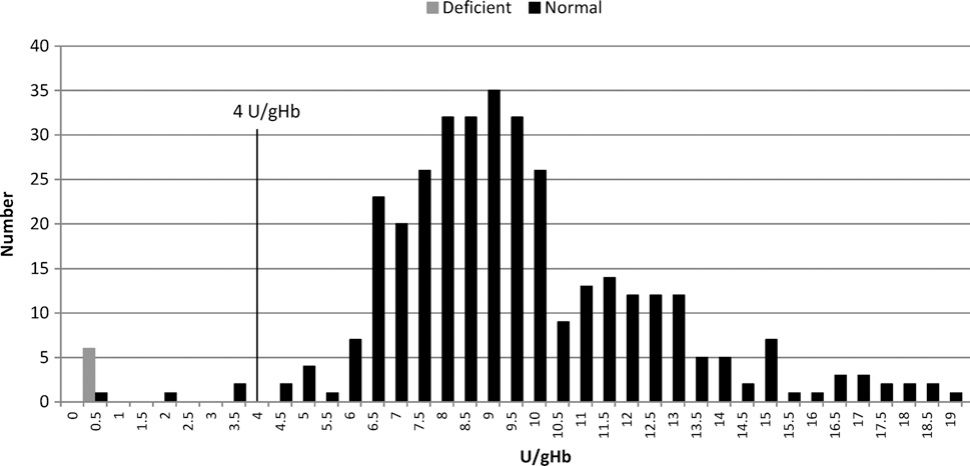
Distribution of G6PD activity according to the BinaxNOW G6PD results.

In total, 401 subjects were screened with the reference test, and from these subjects, 357 subjects were tested with the BinaxNOW G6PD. One subject was found to be *P. vivax*-negative after quality control of slides and hence, excluded from the analysis. Interpretable results were obtained in all 356 subjects tested with both methods: 43 subjects in Brazil, 125 subjects in Peru, 115 subjects in Thailand, and 73 subjects in India (Figure 1

). The study population was mostly men (74.7%), the median age was 33 years old (range = 16–74 years old), geometric mean parasite density was 3,935.4 asexual forms/μL (range = 30–78,800 asexual forms/μL), median Hb was 12.7 g/dL (range = 7.4–16.5 g/dL), and median reticulocytes was 1% (range = 0.2–4.2%). With the quantitative assay, the median G6PD activity was 8.81 U/g Hb, with values ranging from 0.25 to 9.7 U/g Hb in Brazil, from 1.8 to 17.5 U/g Hb in Peru, from 0.05 to 20.19 U/g Hb in Thailand, and from 0.43 to 16.5 U/g Hb in India (Table 1). G6PD activity ranged from 0.05 to 18.81 U/g Hb in men and from 1.86 to 20.19 U/g Hb in women. Compared with the normal G6PD activity values for each reference population, the G6PD activity ranged from 0.43% to 245.9% in men and from 21.8% to 222.7% in women, with a total of 11 (3%) subjects identified as G6PD-deficient (Figure 2

). The deficient subjects were reported from Thailand (*N* = 5), Peru (*N* = 3), India (*N* = 2), and Brazil (*N* = 1); 10 subjects were men, and 1 subject was a woman (Table 1). Sensitivity of the BinaxNOW G6PD to detect deficient subjects was 54.5% (95% CI = 23–83%), and specificity was 100% (95% CI = 98–100%) (Table 2). Four of five subjects incorrectly identified as non-deficient in the BinaxNOW G6PD had enzyme activity in the quantitative assay below the rapid test reported threshold (≤ 4 U/g Hb): 0.4 U/g Hb (male), 1.8 U/g Hb (female), 3.1 U/g Hb (male), and 3.2 U/g Hb (male). The other sample was a male with 4.41 U/g Hb enzyme activity (Figure 3

).

## Discussion

Accurate diagnosis of G6PD deficiency is required to avoid the risk of acute hemolysis associated with 8-aminoquinoline treatment in malaria. In this study, the quantitative spectrophotometric analysis of G6PD activity was set up during a phase 2b clinical trial of the efficacy of primaquine and tafenoquine to support the evaluation of the study entry criteria and used to validate the operational characteristics of the BinaxNOW G6PD test in the field. The point-of-care diagnostic test BinaxNOW G6PD showed a low sensitivity to detect deficient subjects (54.5%) but high specificity (100%) compared with the quantitative spectrophotometric analysis in *P. vivax*-infected subjects. This sensitivity was significantly lower than the values of 98% and 82.6% reported in well-characterized G6PD-deficient and non-deficient blood samples.^14^,^17^ After they were made aware of these false normal results, the BinaxNOW G6PD manufacturers performed additional quality assurance assessment of the tests. They subsequently confirmed that there were no lodged complaints from end users and that there were no notes of false normal results. In addition, they corroborated that quality control release was within acceptable internal specifications, and there were no false normal results for three quality control-deficient samples used to test retained kits from the same batches used in the study (data not shown). Hence, potential explanations for the lower sensitivity in the field study include different characteristics of study populations and different laboratory conditions than those used in the studies by Tinley and others^14^ and LaRue and others.^17^ Other reasons included technical issues, such as difficulty in ensuring that room temperature in the field fell within the range required by the rapid test (18–25°C), subtlety of color change, tight time requirements, and training.

With respect to the study population, blood samples discussed in the works by Tinley and others^14^ and LaRue and others^17^ were from malaria-free individuals in the United States, whereas in this report, we studied *P. vivax*-infected subjects from endemic areas. On one hand, *Plasmodia* infection could potentially influence G6PD activity in RBCs, because it has been shown that *P. falciparum*-infected, G6PD-deficient RBCs have higher G6PD activity than uninfected G6PD-deficient RBCs after a single growth cycle.^18^ This could affect the performance of both the point-of-care test and the quantitative assay. However, the increased G6PD activity (thought to be parasite-derived) is considered small, and in this experiment, the proportion of infected RBCs was several orders of magnitude superior to that usually found in the field. Hence, *Plasmodia* infection *per se* is not expected to hinder the diagnosis of G6PD deficiency. Nevertheless, the methodological challenges of experimentally assessing the behavior of enzymes in human RBCs have been highlighted.^19^ On the other hand, acute hemolysis during a malaria episode and high reticulocyte count may cause a G6PD-deficient subject to be incorrectly diagnosed as normal, because the level of activity in young erythrocytes is higher than in older cells.^6^ In this study, 4.8% (14 of 293) of subjects had > 2.5% reticulocyte count, whereas in the works by Tinley and others^14^ and LaRue and others,^17^ reticulocyte counts were not provided but likely to be in the normal range, because the patients were outpatients. In the future, when comparing the performance of G6PD diagnostic tests in different studies, Hb concentration, reticulocyte count, and RBC volume (MCV) should be considered as potential sources of heterogeneity.

With respect to the technical issues, environmental conditions, such as temperature, affect the performance of different G6PD enzymatic activity assays. This is true for both the spectrophotometric analysis and the BinaxNOW G6PD. For the reference test, spectrophotometers with controlled temperature compartments were used, and results were temperature-corrected. For the BinaxNOW G6PD, the tests were done in the local laboratory facilities, some of which had air conditioning, but thermometers were not always used to guarantee that the ambient temperatures were within the range specified by the manufacturers (18–25°C). Ensuring that ambient temperature is below 25°C will be difficult in many malaria-endemic areas. We cannot rule out direct light inducing a color reaction because of the light sensitivity of reagents in several G6PD activity tests, which may have caused a deficient sample to appear normal, but this is unlikely, because all laboratories were aware of this sensitivity. Likewise, the enzymatic assays are very dependent on time, and hence, the package insert states that the BinaxNOW G6PD should be read at exactly 7 minutes. This tight time requirement could be included as part of the technical considerations for the observed BinaxNOW G6PD performance in the field. Finally, subtlety of color change could affect the visual interpretation of results. Therefore, the need for specialized training may partially explain the low sensitivity found. Of note, we provided no formal training on how to perform the BinaxNOW G6PD, because we considered personnel in the study sites to be experienced laboratory technicians who might run the tests following the available manufacturer's instructions in future real-life conditions.

Despite the low sensitivity found, the predictive values were relatively high. The probability of a subject with a normal result in the BinaxNOW G6PD being, in fact, non-deficient was 99%. Predictive values depend on the prevalence of the disease; thus, as the prevalence of G6PD deficiency increases, the negative predictive value decreases, and the positive predictive value increases. The prevalence of G6PD deficiency in malaria-endemic areas has been predicted to be as high as 23% (e.g., in the Asian-Pacific region); however, high variations within countries are common.^4^,^20^ The recently reported prevalence of G6PD deficiency varying between 0% and 10% in relation to sex and ethnic group in Afghanistan is a case in point.^21^ Likelihood ratios are more useful to interpret the test results, because they do not depend on prevalence of disease. They tell how the test results modify the pre-test probability of disease independent of its prevalence. Because false deficient results were not observed with the BinaxNOW G6PD, the positive likelihood ratio approaches infinity, which indicates that a deficient result in this test confirms the diagnosis of G6PD deficiency. On the contrary, the negative likelihood ratio was above 0.1, which suggests that a non-deficient result in the BinaxNOW G6PD does not rule it out, independent of the prevalence of the G6PD deficiency in the population. Given the potential consequences of treating a G6PD-deficient subject with 8-aminoquinolines, it is more important to rule out diagnosis, and hence, diagnostic tests with high sensitivity and likelihood ratio below 0.1 are required.^22^

One final limitation of this study was the potential that residual misclassification of G6PD-deficient subjects by the quantitative spectrophometric analysis occurred, which would underestimate the sensitivity of the BinaxNOW G6PD. Molecular biology-based methods of diagnosis that detect specific mutations are required to confirm G6PD deficiency, particularly in women as well as in men with unexpected intermediate results (e.g., samples of men with 36.9%, 54.3%, and 51.7% G6PD activity in the spectrophotometric analysis).^6^ Because these subjects failed screening to enter the phase 2b clinical trial, a G6PD genotyping sample was not taken. Reference population G6PD activity values were highly variable, although they were appropriately estimated from only males with normal Hb concentration and reticulocyte count. Inaccurate high cutoff values to define G6PD deficiency in the spectrophotometric analysis would underestimate the sensitivity of the BinaxNOW G6PD. This is probably low, because four of five subjects incorrectly identified as non-deficient by the rapid tests had G6PD activity lower than the 4 IU/g Hb BinaxNOW G6PD-reported detection threshold.^14^ Establishing normal G6PD reference values in the population is challenging. A method that estimates the adjusted median 100% G6PD activity from the study population after excluding those with G6PD activity equal to or less than 10% of the male median has been proposed.^8^ The adjusted median in the study site with the largest sample size (Iquitos) yielded a reference value of 8.45 U/g Hb (cutoff 60% of 5.07 U/g Hb), which was almost identical to the one previously defined (Table 1).

In conclusion, these results suggest that the rapid test BinaxNOW G6PD is unsuitable for the screening of G6PD deficiency in *P. vivax*-infected subjects in endemic areas. Difficulty in ensuring that room temperature in the field fell within the range required by the rapid test (18–25°C) together with the subtlety of the color change and the tight time and specialized training requirements could partially explain the low sensitivity found. Additional development of simple, highly sensitive G6PD deficiency diagnostic tests for routine use in the field is a priority for the safe use of 8-aminoquinolines in the radical cure and elimination of *P. vivax* malaria.

## Figures and Tables

**Table 1:** Results of G6PD activity by the quantitative assay in study samples and corresponding population reference values

Place	Normal reference (cutoff for deficiency) IU/g Hb	Median (range) in study samples IU/g Hb	*N*	Deficient/normal samples	
				Male	Female
Manaus	6.26 (< 3.75)	6.84 (0.25–9.7)	43	1/35	0/7
Iquitos	8.53 (< 5.11)	8.79 (1.86–17.5)	125	2/79	1/43
Bangkok	7.4 (< 4.44)	8.18 (0.38–18.2)	62	3/50	0/9
Mae Sot	11.5 (< 6.9)	12.85 (0.05–20.19)	53	2/44	0/7
Bikaner	5.99 (< 3.6)	6.69 (0.43–12.10)	33	2/20	0/11
Chenaii	11.82 (< 7.1)	12.84 (9.95–13.7)	5	0/3	0/2
Lucknow	11.84 (< 7.1)	9.97 (7.83–16.5)	27	0/19	0/8
Secundarabad	7.9 (< 4.74)	10.52 (9.43–11.69)	8	0/6	0/2

**Table 2:** Measures of performance of the BinaxNOW G6PD

BinaxNOW G6PD	Formula		Quantitative assay	
	Deficient (a + c)	Normal (b + d)	Deficient (*N* = 11)	Normal (*N* = 345)
Deficient	a	b	6	0
Normal	c	d	5	345
Prevalence (P)	(a + c)/(a + b + c + d)		3%	
Sensitivity (Sn)	a/(a + c)		54.5% (95% CI = 23–83)	
Specificity (Sp)	d/(b + d)		100% (95% CI = 98–100)	
Positive predicted value	(Sn × P)/(Sn × P) + [(100 – Sp) × (100 – P)]		100% (95% CI = 42–100)	
Negative predictive value	Sp × (100 – P)/[(100 – Sn) × P] + [Sp × (100 – P)]		99% (95% CI = 97–100)	
Positive likelihood ratio	Sensitivity/(100 – specificity)		∞	
Negative likelihood ratio	(100 – sensitivity)/specificity		0.45 (95% CI = 0.24–0.87)	
